# What have we learned from ten years of trajectory research in low back pain?

**DOI:** 10.1186/s12891-016-1071-2

**Published:** 2016-05-21

**Authors:** Alice Kongsted, Peter Kent, Iben Axen, Aron S. Downie, Kate M. Dunn

**Affiliations:** The Nordic Institute of Chiropractic and Clinical Biomechanics, Odense, Denmark; Department of Sports Science and Clinical Biomechanics, University of Southern Denmark, Campusvej 55, 5230 Odense M, Denmark; Department of Physiotherapy and Exercise Science, Curtin University, Perth, Australia; Intervention and Implementation Research, Institute of Environmental Medicine, Karolinska Institutet, Stockholm, Sweden; The George Institute for Global Health, University of Sydney, Sydney, Australia; Faculty of Science and Engineering, Macquarie University, Sydney, Australia; Arthritis Research UK Primary Care Centre, Institute of Primary Care and Health Sciences, Keele University, Keele, Staffordshire, ST5 5BG UK

## Abstract

**Background:**

Non-specific low back pain (LBP) is often categorised as acute, subacute or chronic by focusing on the duration of the current episode. However, more than twenty years ago this concept was challenged by a recognition that LBP is often an episodic condition. This episodic nature also means that the course of LBP is not well described by an overall population mean. Therefore, studies have investigated if specific LBP trajectories could be identified which better reflect individuals’ course patterns. Following a pioneering study into LBP trajectories published by Dunn et al. in 2006, a number of subsequent studies have also identified LBP trajectories and it is timely to provide an overview of their findings and discuss how insights into these trajectories may be helpful for improving our understanding of LBP and its clinical management.

**Discussion:**

LBP trajectories in adults have been identified by data driven approaches in ten cohorts, and these have consistently demonstrated that different trajectory patterns exist. Despite some differences between studies, common trajectories have been identified across settings and countries, which have associations with a number of patient characteristics from different health domains. One study has demonstrated that in many people such trajectories are stable over several years. LBP trajectories seem to be recognisable by patients, and appealing to clinicians, and we discuss their potential usefulness as prognostic factors, effect moderators, and as a tool to support communication with patients.

**Conclusions:**

Investigations of trajectories underpin the notion that differentiation between acute and chronic LBP is overly simplistic, and we believe it is time to shift from this paradigm to one that focuses on trajectories over time. We suggest that trajectory patterns may represent practical phenotypes of LBP that could improve the clinical dialogue with patients, and might have a potential for supporting clinical decision making, but their usefulness is still underexplored.

## Background

Decisions about health care are traditionally based on a medical diagnosis. However, the most important focus of health care is patient outcomes and, as recently argued by Croft and colleagues, these outcomes are not only determined by disease diagnosis [1]. Sometimes diagnosis actually tells very little about prognosis. Croft and colleagues argue that “prognosis can now provide the framework in which clinicians and researchers organise evidence and information to support decisions about management”, and illustrate this proposition with numerous examples of prognostic factors being fundamental for clinical decisions [1].

Low back pain (LBP) is a health condition in which diagnostic information usually does not tell much about probable future outcomes, as in only a minority of cases can a specific pathoanatomic diagnosis be reached [2]. The majority of LBP is categorised as non-specific LBP and therefore may be better understood and managed within a prognostic framework [1].

Non-specific LBP is often categorised as acute, subacute or chronic focusing on the duration of the present episode [3]. However, more than twenty years ago it was recognised that LBP is often an episodic condition and people who have experienced LBP are likely to also have future episodes [4, 5]. This challenged the concept of acute versus chronic LBP which implies that LBP presents either as unrelated acute episodes or as chronic continuous pain, and an additional limitation of that concept is that it does not differentiate between a recent onset episode experienced for the first time and a recent flare-up of recurrent LBP. Similarly, this categorisation of chronic LBP includes both people with persistent severe pain and people reporting mild symptoms for more than three months.

That LBP often presents as recurrent episodes also implies that the population-averaged course of LBP does not adequately reflect the course experienced by individuals. The averaged course of LBP with early improvement, followed by very little change after 6 to 12 weeks [6, 7], has been translated into a perception of LBP as a condition that is largely unchanged after that time period. However, recognising that LBP comes and goes suggests that it is not the same individuals reporting pain at all time-points.

Despite this, it was not until ten years ago that patterns underlying the averaged course of LBP were investigated. A longitudinal observational study with monthly follow-up measurements showed that distinct trajectories of LBP (patterns of changes in pain over time) could be identified, and indicated that the prognosis of LBP cannot be adequately described in terms of simply recovery or chronicity [8] and also that a population-averaged course of LBP does not adequately reflect the underlying patterns of LBP. Since that pioneering study, trajectory patterns have been identified in a number of cohorts from different settings and countries and using different statistical methods. These have all confirmed that characteristic LBP prognostic groups exist with trajectory patterns that are distinctly different from the population-averaged course. A recent overview of LBP trajectory studies concluded that most people who experience LBP will have trajectories of either persistent or episodic pain rather than one well-defined episode, and suggested that single time-point outcomes are not optimal measures of LBP [9].

In this paper, we provide a summary of the current knowledge on LBP trajectory patterns in adults and describe the main similarities and differences of previous findings. Subsequently, we consider whether LBP trajectory patterns may be useful as a way to define LBP prognostic ‘phenotypes’. Lastly, we discuss how such trajectories may become clinically useful and suggest some areas for future research.

## Discussion

### Which LBP trajectories have been identified?

To our knowledge, LBP trajectories in adults have so far been identified by data driven approaches in ten cohorts [8, 10–18]. In these studies, participants with a main complaint of LBP were followed from three to twelve months with data collection at four to 52 time-points. Outcome measures were LBP intensity, LBP frequency (number of LBP days per week) and activity limitation. Trajectory patterns were identified using either Hierarchical Cluster Analysis, Latent Class Analysis, or Latent Class Growth Analysis (Table 1).

**Table 1:** Overview of ten studies in which LBP trajectories have been identified by data-driven approaches

Author Setting Sample size	Design	Timing and duration of follow-up	Measurement tool	Outcome measure^a^	Clustering method	Identified clusters Label (% of cohort)
Dunn Primary care General practice *n* = 342 (2001–03) [8] *n* = 155 (2009–10) [10]	Observational	Monthly for 6 months	Questionnaires	LBP Intensity 3-cat.	Latent Class Analysis	2001-03 cohort Persistent mild 31 % Recovering 30 % Severe chronic 21 % Fluctuating 13 % 2009-10 cohort Persistent mild 37 % No or occasional 31 % Persistent severe 21 % Fluctuating 11 %
Axen Primary care [12] Chiropractic practice *n* = 176	Observational	Weekly for 6 months	Text messaging	LBP Frequency 0–7	Hierarchical Cluster informed by spline regression (intercept, slopes, knot)	Typical [improve markedly during 4 weeks] 41 % Stable [mild] 24 % Slow improvement 15 % Fast improvement 13 % Not classified 6 %
Kongsted Primary care [11] General practice + Chiropractic practice *n* = 1082	Observational	Weekly for 12 months	Text messaging	LBP Intensity 0–10 LBP Intensity 3-cat. LBP Frequency 0–7 LBP Frequency 3-cat.	Latent Class Analysis Latent Class Growth Analysis	^b^Mild episodic 29 % Recovery 26 % Moderate/ severe 20 % Improvement w/ relapse 13 % Slow improvement 12 %
Macedo Primary care + Secondary care [16] General practice + outpatient clinic (≥3 months duration) *n* = 155	RCT	Monthly for 12 months	Text messaging	LBP Intensity 0–10	Hierarchical Cluster informed by linear regression (deviations from line)	Non-fluctuating 87 % recovering mild 54 % persistent moderate 58 % severe chronic 17 % Fluctuating 13 %
Chen Workers on sick leave [13] *n* = 678	Observational	Week 4, 10, 16,52	Interview	LBP Intensity 0–10	Hierarchical Cluster informed by linear regression (slope)	Continuous high 42 % Fluctuating 33 % Large reduction 12 % Moderate reduction 12 % Increasing 1 %
Tamcan Population-based [14] *n* = 305	Observational	Weekly for 12 months	Diary	LBP Intensity 3-cat.	Latent Class Analysis	Moderate 35 % Fluctuating 34 % Mild 20 % Severe 10 %
Kent Secondary care [15] Outpatient clinic *n* = 322	RCT	Fortnightly for 12 months Monthly for 12 months	Test messaging	LBP Frequency 0–7	Two-step cluster	Fortnightly outcomes^c^ Severe persistent 42 % Moderate 33 % Severe fluctuating 25 % Monthly outcomes Severe 62 % Moderate 38 %
Deyo Primary care + emergency care [17] Age >65 years *n* = 3929	Observational	Month 3, 6, 12	Questionnaire or phone	LBP intensity 0–10 Activity limitation	Latent Class Analysis	Pain intensity Moderate – High 36 % Low – Moderate 31 % High 13 % Moderate –Recovery 7 % Severe – Recovery 7 % Low 6 % Activity limitation Moderate – High 32 % Low – Moderate 25 % Low 19 % High 19 % Recovery 6 %
Downie Primary care [18] General practice (<6 weeks duration of LBP) *N* = 1585	RCT	Week 1, 2, 4, 12	Recorded in a booklet - transcribed by phone	LBP intensity	Latent Class Growth Analysis	Rapid recovery 36 % Recovery by week 12 34 % Incomplete recovery 14 % Fluctuating pain 11 % Persistent high pain 5 %

^a^LBP Frequency = Number of days with LBP last week

^b^The study presented 12 different models with from five to twelve trajectory patterns identified. The example was based on categorical LBP intensity

^c^Trajectories were named for the purpose of this paper. In the paper they were labeled with numbers

From two to twelve discrete LBP trajectory patterns have been identified in these published studies. Two studies did head-to-head comparisons of results of Latent Class Analysis when changing the number of outcome time points [15] or the type of outcome measure [11]. A general observation from these two studies was that an increased numbers of trajectory patterns primarily resulted from a more detailed separation of trajectories rather than identification of new substantively different patterns, for example the identification of rapid and slow recovery instead of just one common recovery pattern.

**Fig. 1:**
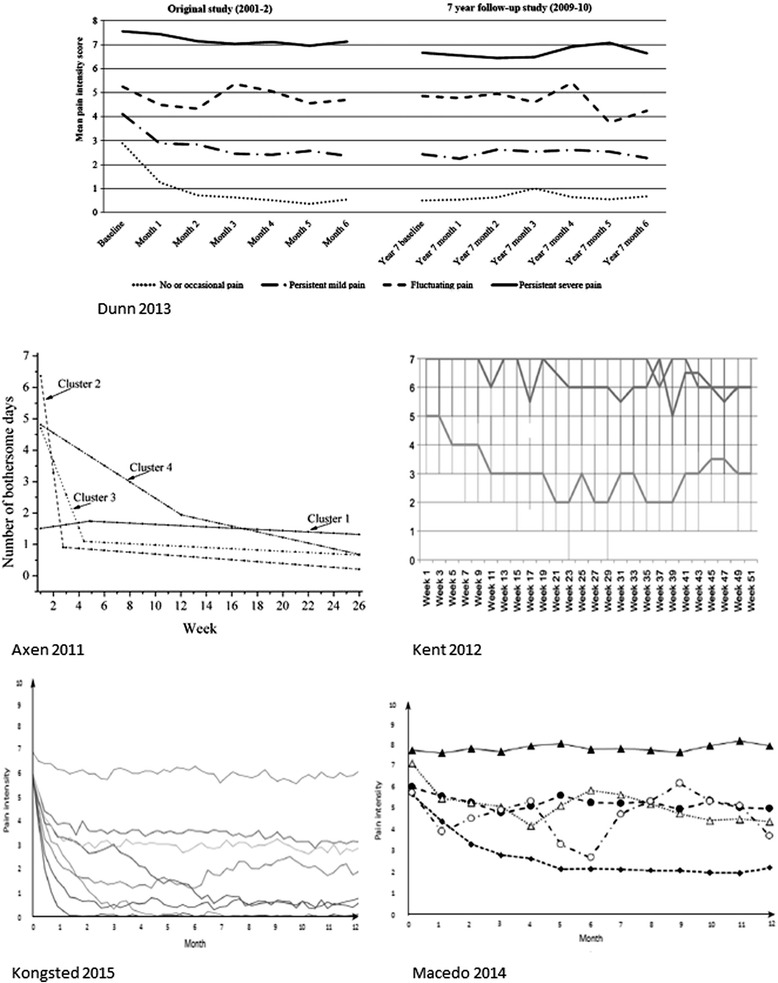
Illustrations of trajectories identified in five previously published studies. Each trajectory is represented by mean values of the subgroup. Dunn 2013 is from [10], Axen 2011 is from [12], Kent 2012 is from [15] and Kongsted 2015 is from [11]. These papers were published as open access and therefore the authors hold the copyrights of the reprinted illustrations. Macedo [16]: The illustration was not published in the original paper and was provided by the author

Four or five trajectory patterns were identified as the optimal number of trajectory patterns in most cohorts (Table 1). Two studies identified patterns that mainly differed in severity and less in the course patterns [8, 14], whereas the other studies also observed differences in course patterns seen as non-parallel and crossing trajectory lines in Fig. 1. All studies identified a pattern of *recovery*, except the study by Macedo and colleagues, which included only LBP patients with at least three months duration. The studies also described a trajectory pattern of *persistent severe LBP*, apart from the study by Axen and colleagues which excluded patients with unchanging pain, as this was required due to their choice of analytic approach. In addition, all studies identified patterns that were neither a rapid recovery nor persistent severe pain. Different patterns of improvement in the very early course were mainly observed in the studies that included many patients with recent onset pain [11, 12, 18]. In addition, most studies described *fluctuating patterns* characterised by LBP of alternating intensity and/or by LBP episodes with periods of no pain. It should be noted that, although the clustering techniques aim at reducing the within-class variability, individual course patterns within the latent classes will differ. So while the trajectories in Fig. 1 are illustrated by subgroup means they will not reflect the more fluctuating nature of LBP in some individuals.

The proportion of patients in each trajectory pattern differed within different settings, as did the number of identified patterns (Table 1). However, a consistent finding was that the majority of patients in primary care populations had patterns of pain of mild intensity or had infrequent LBP (a few days per week), whereas approximately one in five patients had persistent severe pain. Trajectory patterns were reported as displaying fluctuating pain in less than 15 % of the cohorts when classification was based on Latent Class Analyses of monthly outcome measures [8, 10, 16] but more frequently (25–34 %) with weekly or fortnightly measures [11, 14, 15]. One study with weekly outcome measures used a linear analytic approach that did not separate between stable and fluctuating patterns [12].

In summary, studies of LBP trajectories all found that the course of LBP originates from more than one underlying distribution and therefore it is clear that the population-averaged course of LBP does not represent individuals’ course. We observe that different trajectory patterns exist and that common trajectory patterns are identified across settings and countries. It is also apparent that there are differences across studies. To some degree this seems to be a result of including study participants with different episode duration, of measuring with different frequencies, and of using different statistical approaches. Other cohort characteristics and the potential effects of treatment response may also have influenced this diversity. Furthermore, the number of patterns described was, to some extent, a result of the subjective decisions required with these types of analytic methods. For example, one study decided a priori to look for four subgroups, whereas others have used different statistically-driven stopping rules that inform the choice of which cluster structure best represented the data. These stopping rules do not indicate *one* clearly optimal cluster solution and choosing the number of subgroups based on the best statistical fit of data may not identify the most clinically useful number of subgroups. Therefore decisions about the best cluster solution are made using a combination of statistical measures and clinical interpretation of the cluster solutions. Sometimes, a higher number of subgroups revealed some potentially interesting trajectory patterns, but it is still unknown if this is important for better understanding of LBP or its clinical management.

Given the relative consistency observed across studies, despite differences in study methods, we believe it likely that further studies using data-driven identification of LBP trajectories would replicate similar patterns to those already observed. The question remains whether additional exploratory studies of this kind would increase our understanding of the course of LBP. It is possible that further explorative studies in more diverse samples, such as children or old people, in developing countries or in selected clinical groups such as surgically treated patients, may reveal other trajectory patterns. However, in our opinion it is unlikely that new studies of trajectories in similar populations to those already studied would change the current picture of LBP.

### Could trajectories identify useful phenotypes of LBP?

There is considerable interest in whether the identification of prognostic subgroups of LBP could improve our understanding of LBP and assist the making of better decisions about clinical management. For trajectory subgroups to be clinically useful phenotypes of LBP, ideally they would be relatively stable over time, meaningful for clinicians and patients, and provide guidance for treatment decisions.

One study has investigated the stability of LBP trajectories over time by following the same cohort over two six-month periods that were seven years apart [10]. The results suggested that the majority of adults in their thirties to fifties remain in a particular LBP trajectory. Only patients with a fluctuating course pattern were very likely to have a shift of trajectory pattern, typically into patterns of either mild ongoing or severe ongoing LBP. If more widely confirmed, this would imply that most patients with LBP have a particular pattern of LBP, and perhaps that the most likely opportunity for shifting patients from one trajectory to another is in patients presenting with a fluctuating pattern.

To be useful, LBP phenotypes also need to make clinical sense. The potential for clinical use is still unexplored, but patients seem able to retrospectively identify their own trajectory pattern when presented with illustrations of the general trajectory patterns [19]. Similarly, unpublished qualitative research from within our group indicates that patients’ retrospective appraisal of their trajectory pattern aligns well with the way researchers interpret their quantitatively-derived trajectories. From our experience, patients find the trajectories meaningful and, although not yet supported by empirical evidence, trajectories may be an opportunity for patients to better understand their condition.

It is widely recognised that LBP should be understood within a biopsychosocial framework [20], and unidimensional LBP trajectory patterns are probably only potentially important if associated with other key patient characteristics. Published studies have consistently found that the identified trajectories are associated with a number of patient characteristics that had not been used as part of the trajectory subgroup formation. This is reassuring because it confirms that the trajectory subgroups actually represent different patient profiles. There is evidence that trajectory patterns are associated with factors across health domains including activity limitation [8, 11, 14, 16–18], work participation [8, 14], LBP history [8, 11–14, 17, 18], leg pain [8, 11, 18], depression/anxiety [8, 10, 11], recovery expectations [11, 17, 18] and general health/comorbidity [10, 12–14, 17, 18]. Also catastrophizing [8], compensable LBP [18] and the outcomes of health care utilisation [8] and global perceived effect [11] were associated with LBP trajectories in single studies that investigated these factors. Trajectories were not associated with age in broad clinical populations [8, 10–12, 18], whereas higher age was associated with more severe trajectory patterns in non-clinical cohorts and in the elderly [13, 14, 17]. Trajectories were generally not associated with sex, whereas investigations of a relationship with education [11, 13, 14, 17] and sleep disturbances [10, 18] have shown mixed results.

Even though the approach to subgroup identification differed, similar patient profiles have generally been identified across studies. Consistently, patients with LBP trajectories of mild or transient pain had the least activity limitation and least psychological issues, whereas patterns of high intensity pain were associated with more constant pain, higher levels of disability, depression, anxiety, sick leave and other indicators of poor health and quality of life.

We suggest that the capacity of LBP trajectories to be clinically useful LBP phenotypes deserves attention as this simple unidimensional approach results in subgroups that appear consistent across cohorts and over time, are recognisable to patients, and differ on a number of key clinical characteristics across health domains. It would be interesting to investigate whether LBP subgroups identified from their pain trajectory are as useful for the classification of LBP patients as existing multidimensional prognostic tools.

### What might be the use of LBP trajectories?

It is not clear what the use of trajectory patterns may be, but the identification of LBP trajectories has underpinned suggestions by previous researchers [21], that the division of LBP into only acute or chronic duration is a limited, and limiting, classification approach. The evidence is consistent that distinct trajectory subgroups exist which allow more subtle and precise classification. It is timely for these findings to facilitate a paradigm shift.

The traditional distinction between acute and chronic LBP, and the approach to LBP as a condition with an outcome of simple recovery/non-recovery, are reflected in LBP trials and clinical guidelines focusing on patient cohorts with acute (recent onset) or chronic (long duration) LBP, often with single time-point measurements of short- and long-term effects. The emerging knowledge about the course of LBP suggests that it might be useful to differentiate between treatments directed at an episode of increased LBP (a ‘flare-up’) and interventions intended for managing patients’ long-term LBP patterns. This would further imply that effects related to treatment of flare-ups are best measured by use of short-term outcome measures, whereas interventions directed at long-term patterns need to be evaluated by outcome measures that reflect trajectory patterns. In the design of future clinical trials, it may be beneficial to consider if a more distinct focus on treatment goals (overcoming a flare-up or trying to shift a trajectory) and the use of trajectories to identify homogenous groups for inclusion to the trial would provide more clinically useful insights. An example would be the identification of people with episodic pain who would be candidates for secondary prevention.

It has been advocated that repeated outcome measures rather than single time-point measures are needed in LBP [9], and trajectory patterns may appear attractive as outcome measures. However, the potential for altering a trajectory is still unknown and the use of trajectories as outcome measures would require that they be validated as such. How that might be performed is not straight forward, for example there is no obvious reference standard for assessing responsiveness.

If LBP trajectories reflect different phenotypes of LBP, their relationship to treatment response might differ. Patients with different trajectory patterns may respond differently to a given treatment, or may benefit from different treatments. This would be conceptualised as LBP trajectories being potential treatment effect moderators. It is also possible that the phenomenon observed in some clinical cohorts of differentiation into trajectory patterns very soon after treatment was initiated could be a key to identify yet unrevealed effect moderators responsible for the observed differences. Also, we speculate that trajectory patterns may be more suitable phenotypes for geneticists investigating LBP than the current, and maybe simplistic, definitions of LBP.

Furthermore, the identification of LBP trajectories suggests a way to record patients’ LBP history, as an alternative to simply recording any previous episodes. Potentially, patient self-report of previous LBP trajectories could be a more accurate predictor of future LBP than measures such as episode duration and number of previous episodes, and this should be tested. Another possible clinical use of identified trajectories is as a tool to help patients understand the nature and prognosis of their condition and as a reference for discussing treatment goals.

The only clinical assessment instrument that we know of which currently uses trajectory patterns is the PainDETECT questionnaire. This questionnaire is designed to identify people that might have neuropathic pain based on their self-reported pain characteristics [22]. It includes a visual representation of four trajectory patterns from which a patient can select the pattern closest to their pain experience. However, as no information on how these PainDETECT patterns were derived is reported and as they do not represent the common trajectory patterns identified in broad LBP cohorts, they have unknown utility for LBP patients.

### Areas for future research into trajectories

Rather than conducting additional studies aiming at the identification of latent LBP trajectories in adults, we think a better research priority is to investigate if the patterns already described can be confirmed in new cohorts and also investigate if trajectories are similar across musculoskeletal pain conditions. Methods exist by which trajectories identified in one cohort can be applied to a new cohort and the model fit calculated. Some similarities to the trajectory patterns observed in LBP have been shown in knee pain [23, 24] and there is also preliminary evidence from short-term follow-up in neck pain [25]. Also, it would be useful to have confirmatory evidence that people tend to stay in the same trajectory for many years and at what age trajectory phenotypes are established. Some evidence exists that the findings of distinct LBP trajectories seen in adults also apply to adolescents [26], but LBP trajectories in children and adolescents remains an area of limited investigation. The number of data points needed to confirm the observed trajectories depends on the intended level of detail. Some trajectories can be identified from monthly collected data, whereas differentiation of trajectories with different rates of improvement in the early phase of a new episode would probably require weekly data for at least five weeks. Conversely, trajectories based on daily pain data may be computationally intensive without posing any obvious advantage over less frequent data collection. It is still not known if frequent follow-ups may in itself affect recovery rates, but outcomes in studies with frequent follow-ups are not obviously different from what has generally been observed in LBP.

To start understanding the possible importance of trajectories, it would be revealing to determine whether successful treatment can shift a patient’s trajectory to a more optimal one or help a patient return to their usual trajectory. This knowledge would inform a more nuanced understanding of what realistic treatment goals in LBP might be. Furthermore, it would be helpful to determine how long after first onset of pain a patient’s future trajectory can be accurately predicted, and whether using a patient’s previous trajectory as prognostic marker increases the accuracy of prediction. As touched upon previously, it would also be highly relevant to investigate whether LBP trajectories identify phenotypes of LBP that benefit from different care pathways.

Finally, the identification of trajectories of ongoing LBP points towards the relevance of investigating whether groups of LBP patients exist that benefit from a management approach which takes the life-course of LBP into account, rather than dealing with each occurrence or flare-up of LBP as an isolated event. Such an approach has been found useful in other health conditions, such as in the care of diabetes.

Research questions such as confirming observed patterns will require that data are collected repeatedly by questionnaires, diaries or SMS as in the previously conducted studies, whereas having people describing their previous trajectory may be supported by graphic presentations of LBP patterns (discussed further below). Furthermore, the lines of inquiry outlined above might be augmented by using qualitative approaches to explore patient interpretations of trajectory patterns and their perspective of including trajectory patterns as part of the clinical encounter.

### Moving forward

**Fig. 2:**
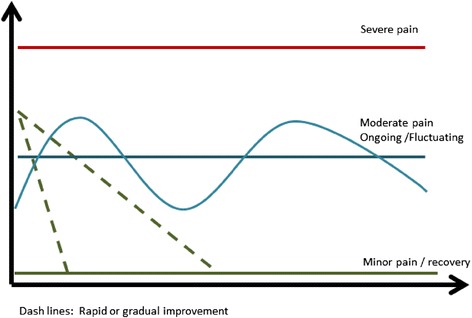
Mean LBP intensity of simplified principal trajectory patterns

**Fig. 3:**
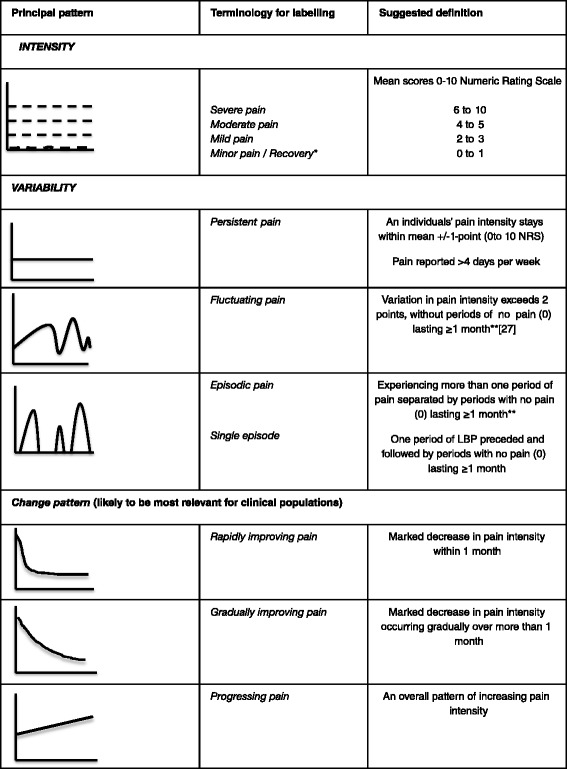
Principal trajectories with suggested labelling. Labels combine a descriptor of *intensity*, *variability* and *change pattern*. The suggested definitions are mainly based on interpretive consensus among the authors about commonly observed trajectories and therefore should be altered as evidence for other definitions may emerge. *The term ‘recovery’ would be suitable for groups that initially present with pain. **Using the definition of episodes suggested by de Vet et al. [27]

**Fig. 4:**
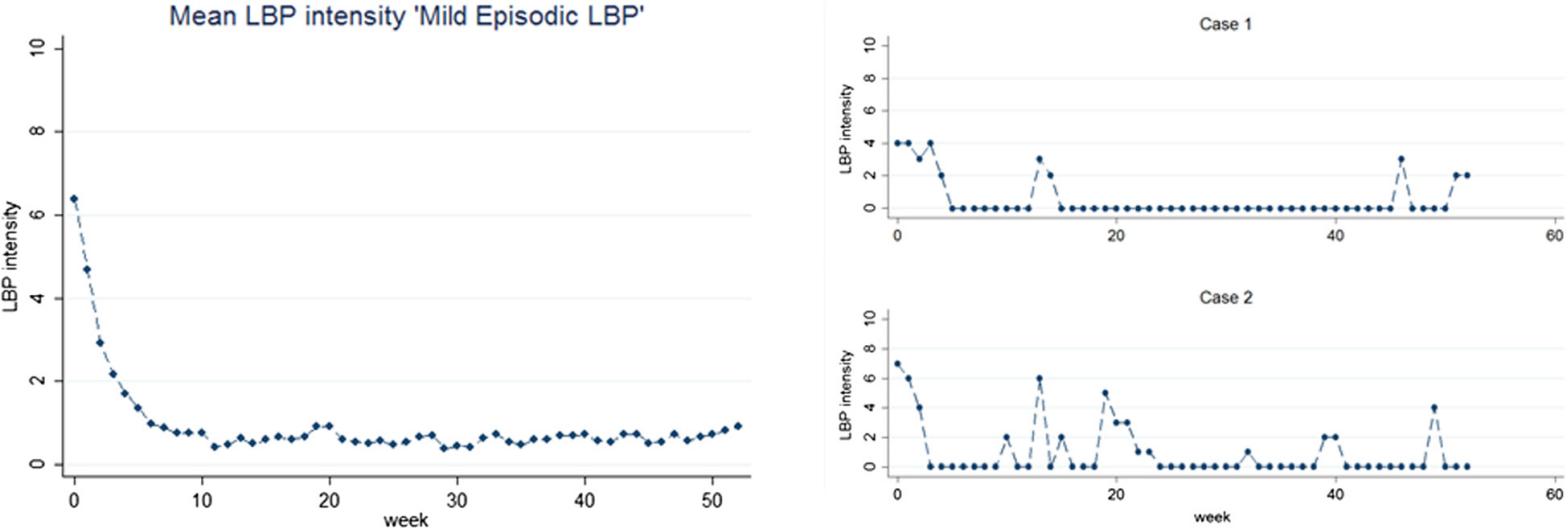
Example of trajectory labelling: Mild episodic LBP. The image on the left side illustrates the mean LBP intensity of patients in a trajectory subgroup labelled ‘Mild episodic LBP’. The images on the right side show two examples of individual trajectory patterns in this subgroup

One first step to move this field of research forwards would be to standardise which principal trajectory patterns to look for and how these are labelled. This could lead to standardised ways in which these are measured to allow consistency in comparisons across studies. For instance, the use of standardised trajectories would be relevant for defining ways to use patients’ self-reports of trajectory patterns and when exploring how trajectories might be used in communication with patients. Also, in the identification of trajectory patterns in new cohorts, it would be useful to be able to describe findings in standardised ways. As a starting point, we suggest that future studies look for groups of people with the principal trajectories illustrated in Fig. 2. Further we suggest that the labelling of trajectory patterns include descriptions of pain intensity, variability over time and (at least for clinical populations) speed of improvement (Fig. 3); for example, ‘rapid improvement to a level of mild fluctuating pain’. Future studies may demonstrate that the suggested definitions in Fig. 3 are not consistent with actual observed patterns, and they therefore should be refined as new knowledge emerges. Since methods such as Latent Class Analysis allow for variation within subgroups, we suggest it is useful to label subgroups in ways that capture the presentation of individuals in that subgroup and not only the subgroup mean. For example, the mean pain intensity of the trajectory pattern named *mild episodic pain* by Kongsted et al. [11] was relatively stable, and the descriptive label indicated that most individuals belonging to this class reported periods of pain separated by periods of no pain (Fig. 4). Naming trajectories could also be valuable in the communication with patients; this could be explored in qualitative studies.

## Conclusions

Studies of LBP trajectory patterns have consistently demonstrated that the course of LBP is not optimally described by the overall population average. Instead, some distinct trajectory patterns have been identified across cohorts and settings. The identified trajectories illustrate that, for most patients, LBP is not a condition from which they either experience a rapid recovery or develop chronic severe pain. Rather LBP is a condition of persistent or fluctuating pain of low or medium intensity. This underpins previous arguments that the differentiation between acute and chronic LBP is overly simplistic and restricting, and we believe it is time to shift from the paradigm of acute and chronic LBP to one that focuses on trajectories over time. Using acute and chronic to categorise LBP implies that the duration of pain is a main discriminator, but trajectory research illustrate that “acute LBP” is often an episode or a flare-up in an ongoing (chronic) condition and also that “chronic LBP” (LBP lasting for more than 3 months) includes very different conditions.

It appears that trajectory patterns might be relatively stable in individuals over time. That stability, combined with trajectories being associated with a large number of other patient characteristics across health domains, suggests that trajectory patterns may represent practical phenotypes of LBP that could improve the clinical dialogue with patients and might have a potential for supporting clinical decision-making.

We argue that additional data-driven trajectory identification in populations similar to those previously studied are not likely to reveal other LBP trajectories that would further shift our understanding of the course of LBP. We suggest that the investigation of LBP trajectories as prognostic markers, aids to patient dialogue, potential treatment effect modifiers and perhaps at some point as outcome measures, is an opportunity to gain a better understanding of this clinically challenging condition. LBP trajectories appear to make sense to patients, clinicians and researchers, but their usefulness is still unproven.

## Ethics and consent to participate

Not applicable as no subjects involved.

## Consent to publish

Not applicable.

## Availability of data and materials

Not applicable.

## Abbreviation

LBP: low back pain
